# 

**Published:** 1896-02-15

**Authors:** 


					No. 490. Vol. XIX.
Saturday,
Feb. 15th, 1896.
WITH EXTRA NURSING SUPPLEMENT.
[Registered at the General Post Office as a Newspaper for Monthly Parts, 6d.; post free, 8d.
transmission in the United Kingdom and Abroad.] Ditto per Annmn.8s.6d., post free.

				

